# Was ist gesichert in der Therapie der metabolischen Azidose bei chronischer Nierenkrankheit?

**DOI:** 10.1007/s00108-024-01806-z

**Published:** 2024-11-08

**Authors:** Alexander Ritter, Christian Kuhn, Nilufar Mohebbi

**Affiliations:** 1 Klinik für Nephrologie und Transplantationsmedizin, Kantonsspital St. Gallen, St. Gallen, Schweiz; 2 Klinik für Nephrologie, Universitätsspital Zürich, Zürich, Schweiz; 3 Praxis und Dialysezentrum Zürich-City, Stockerstrasse 50, Zürich, Schweiz; 4 Universität Zürich, Zürich, Schweiz

**Keywords:** Knochenstoffwechsel, Alkalitherapie, Muskelabbau, Kardiovaskuläre Erkrankungen, Schilddrüsenfunktion, Bone metabolism, Alkali treatment, Muscle wasting, Cardiovascular diseases, Thyroid function

## Abstract

Eine präzise Regulation des Säure-Basen-Haushalts ist für viele Organe und physiologische Prozesse essenziell. Säureretention und metabolische Azidose (MA) sind häufige Komplikationen bei chronischer Nierenkrankheit („chronic kidney disease“ [CKD]) und treten auch nach Nierentransplantation auf. Neben diätetischen Maßnahmen kommen medikamentöse Therapien zur Azidosekorrektur zum Einsatz, mit Natrium(hydrogen)karbonat als am häufigsten eingesetzter Substanz. Mehrere Studien konnten einen positiven Effekt einer Azidosekorrektur auf die CKD-Progression aufzeigen. Die Studienresultate sind jedoch nicht einheitlich und es ist von eher kleineren Behandlungseffekten auszugehen. Nach Nierentransplantation konnte bisher keine positive Wirkung auf die Transplantatfunktion nachgewiesen werden. Die MA ist mit einer eingeschränkten Knochenqualität assoziiert, wobei Alkaliinterventionsstudien bislang einen positiven Effekt auf Marker des Knochenstoffwechsels, nicht jedoch auf die Knochendichte gezeigt haben. Die MA ist mit einer erhöhten kardiovaskulären Ereignisrate assoziiert, Interventionsstudien mit harten kardiovaskulären Endpunkten fehlen jedoch bis dato. Eine Interventionsstudie mit jedoch wesentlichen Limitationen konnte einen positiven Effekt einer Alkalitherapie auf die Mortalität zeigen. Eine Azidosekorrektur scheint sich positiv auf den Protein- und Muskelkatabolismus auszuwirken, wobei eine Verbesserung der körperlichen Leistungsfähigkeit in einer geriatrischen Population nicht gezeigt werden konnte. Bezüglich der endokrinologischen Effekte einer Alkalitherapie existieren nur sehr wenige Studien. Hier zeigten sich ein günstiger Effekt auf den Glukosestoffwechsel und ein möglicher Nutzen in Bezug auf die Schilddrüsenfunktion bei prädialytischen Patienten mit CKD. Aufgrund der insgesamt eher geringen bis moderaten Evidenz für den Nutzen einer Alkalitherapie sowie angesichts der teilweise widersprüchlichen Studienlage wird in den aktualisierten Leitlinien von Kidney Disease: Improving Global Outcomes (KDIGO) die Empfehlung für Erwachsene abgeschwächt und eine Alkalibehandlung vorgeschlagen, um ein Serumbikarbonat < 18 mmol/l (bislang < 22 mmol/l) und die damit verbundenen Komplikationen zu vermeiden.

## Abstract (en)

Precise regulation of the acid-base balance is essential for the functioning of various organs and physiological processes. Acid retention and metabolic acidosis (MA) are frequent complications of chronic kidney disease (CKD) and can also occur following kidney transplantation. In addition to dietary modifications, pharmacological interventions, most notably sodium bicarbonate, are employed to correct MA. While several studies have reported a beneficial effect of MA correction on the progression of CKD, the results remain inconsistent and the magnitude of the treatment effect may be limited. Importantly, no beneficial effect on graft function has been demonstrated after kidney transplantation. The MA is associated with impaired bone quality and although alkali treatment has generally shown positive effects on markers of bone metabolism, consistent changes in bone density have not been observed. Additionally, MA is linked to an increased incidence of cardiovascular events but so far there is a lack of interventional studies with definitive cardiovascular endpoints. Sodium bicarbonate may lead to sodium retention, potentially increasing blood pressure, although the data on this are inconclusive. One interventional study with notable limitations reported a positive effect of alkali treatment on mortality. Correction of MA has been suggested to positively impact protein and muscle catabolism, although no improvement in physical performance was observed in a geriatric population. Limited studies exist on the endocrinological effects of alkali treatment but these indicate a favorable impact on glucose metabolism and potential benefits for thyroid function in predialysis CKD patients. Given the overall low to moderate level of evidence supporting the benefits of alkali treatment, the current guidelines from Kidney Disease: Improving Global Outcomes (KDIGO) propose alkali treatment to prevent serum bicarbonate levels < 18 mmol/l (prior < 22 mmol/l) in adults and the resulting complications.

Die präzise Kontrolle des Säure-Basen-Haushalts ist für viele physiologische Prozesse essenziell, dabei spielen die Nieren eine wesentliche Rolle [21]. Bei chronischer Nierenkrankheit („chronic kidney disease“ [CKD]) kommt es zur Säureretention und folglich zur metabolischen Azidose (MA; übliche Definition: Serumbikarbonat < 22 mmol/l; bis 9 % im CKD-Stadium G3b, bis 37 % im CKD-Stadium G4; [16]). Wegen zusätzlicher Faktoren, welche die Säureausscheidung behindern, wie der Einnahme von Calcineurininhibitoren, ist die Prävalenz nach Nierentransplantation noch höher [31]. Potenzielle negative Auswirkungen einer MA bei CKD sind in Abb. 1 zusammengefasst.

**Abb. 1:**
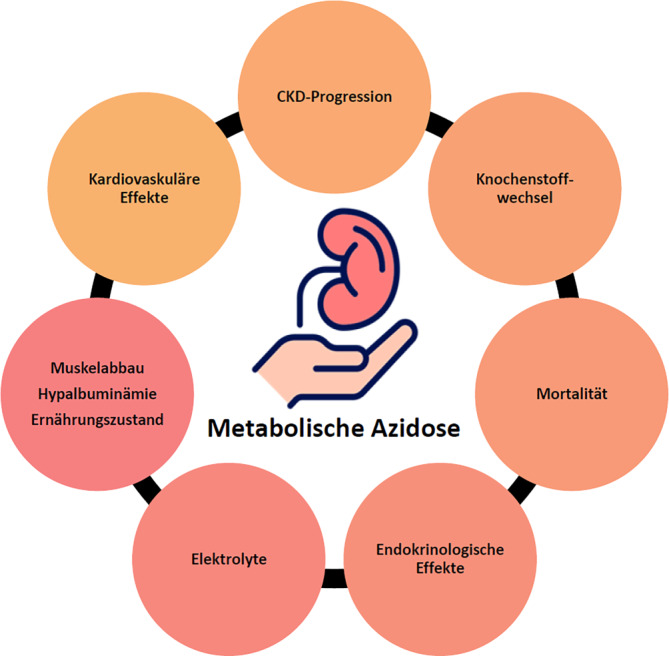
Potenzielle negative Auswirkungen einer metabolischen Azidose bei CKD. *CKD* „chronic kidney disease“ (chronische Nierenkrankheit). (Erstellt mit Nierenbild von https://www.flaticon.com/authors/bsd)

Der vorliegende Beitrag fasst die aktuelle Evidenz zu MA und Alkalitherapien in Bezug auf verschiedene Organsysteme zusammen, ohne Anspruch auf Vollständigkeit. Der Fokus liegt auf randomisierte, kontrollierte Studien (RCT).

## Alkalitherapien

Verschiedene Formen der Alkalitherapie wurden in RCT untersucht, wobei ausnahmslos die orale Verabreichung im Fokus stand. Untersucht wurden sowohl medikamentöse als auch diätetische Alkalitherapien. Allerdings ist die Studienlage und somit auch die Evidenz bezüglich der diätetischen Alkalitherapie stark limitiert. Die am häufigsten eingesetzte Substanz ist Natriumhydrogenkarbonat (chemische Formel: NaHCO_3,_ im Englischen häufig mit „sodium bicarbonate“/Natriumbikarbonat gleichgesetzt). Als Alternative existieren je nach Land weitere Präparate, beispielsweise Kaliumcitrat oder Kalium-Natrium-Hydrogencitrat (Tab. 1). Citrat wird in der Leber zu Bikarbonat metabolisiert. Bei kaliumhaltigen Präparaten ist darauf zu achten, dass bei fortgeschrittener Nierenkrankheit keine Hyperkaliämie auftritt.

**Tab. 1:** Verfügbare pharmakologische Alkalitherapien

Darreichungsform	Substanz				
	Natriumhydrogenkarbonat	Kaliumcitrat	Kalium-Natrium-Hydrogencitrat	Natriumcitrat	Natriumbikarbonat
Kapsel	x	–	–	–	–
Granulat	–	–	x	–	–
Flüssig	–	–	–	x	x
Tablette	–	x	–	–	–

Eine Herausforderung stellt die Alkalitherapie bei Kindern oder Patienten mit Schluckstörung dar, da die Einnahme relativ großer Tabletten oder Kapseln schwierig sein kann. In solchen Fällen kann eine flüssige Darreichungsform wie Shol-Lösung (Natriumcitrat-Citronensäure-Lösung) oder verdünnte intravenöse Natriumhydrogenkarbonatlösung (1-molar) verwendet werden [35]. Seit 2021 ist in einigen europäischen Ländern auch Kaliumcitrat in Kombination mit Kaliumhydrogenkarbonat als Granulat erhältlich [2].

## Progression der chronischen Nierenkrankheit

In den letzten Jahren wurden mehrere RCT unterschiedlicher Größe, Studiendauer und Qualität bei Patienten mit verschiedenen CKD-Stadien und Azidosegraden durchgeführt, um die Wirkung einer Alkalitherapie auf die Nierenfunktion zu untersuchen (Tab. 2), da die MA insbesondere eine renale Fibrose zu begünstigen scheint [21]. Eingesetzt wurde dabei meist Natriumhydrogenkarbonat, aber auch Natriumcitrat oder diätetische Maßnahmen. Drei aktuellere Metaanalysen umfassen mehrheitlich kleine bis mittelgroße Studien mit und ohne Placebokontrolle. Die Ergebnisse lieferten nur eine geringe Evidenz dafür, dass die Alkalitherapie die Progression der CKD verlangsamt und/oder das Risiko eines Nierenversagens verringert [15, 27, 37].

**Tab. 2:** Evidenzlage in Bezug auf Interventionsstudien mit Alkalitherapie bei CKD und metabolischer Azidose

Organsystem/Effekt	Endpunkt(e)	Anzahl Studien (1, 2–5, > 5)	Studienteilnehmer (< 50, 50–200, > 200)	Zusammenfassung der Evidenz
Nierenfunktion	eGFR, Kreatininclearance, Nierenversagen	> 5	< 50 bis > 200	Limitierte Evidenz für Behandlungsnutzen bei CKD; keine Evidenz für Behandlungsnutzen nach Nierentransplantation
Knochen	Knochenstoffwechselparameter, Knochenhistologie, Knochendichte	> 5	< 50 bis 50–200	Limitierte Evidenz für Behandlungsnutzen bezüglich Knochenhistologie und Knochenstoffwechselparameter, keine klare Evidenz für Einfluss auf Knochendichte bei CKD Limitierte Evidenz für Behandlungsnutzen bezüglich Knochenhistologie und Knochenstoffwechselparameter, aber nicht bezüglich Knochendichte nach Nierentransplantation
Gesamtmortalität	–	> 5	< 50 bis > 200	In der größten Studie Reduktion der Mortalität (Odds Ratio 0,45; 95 %-KI 0,22–0,90), in anderen Studien jedoch nicht reproduzierbar, inklusive Metaanalyse
Muskelabbau	Serumalbumin	> 5	< 50 bis > 200	Signifikante Zunahme des Serumalbumins (Metaanalyse: SMD 0,25; 95 %-KI 0,14–0,36)
	Gleichgewicht, Gehgeschwindigkeit oder Beinkraft	1	> 200	Kein Unterschied
	„Lean body mass“ (Oberarmumfang)	2–5	< 50 bis > 200	Signifikante Zunahme (Metaanalyse: SMD 0,23; 95 %-KI 0,08–0,38)

*CKD* „chronic kidney disease“ (chronische Nierenkrankheit), *eGFR* geschätzte glomeruläre Filtrationsrate, *KI* Konfidenzintervall, *SMD* standardisierte Mittelwertdifferenz

Die größte Studie (UBI; multizentrisch; 740 Patienten mit CKD-Stadium G3–5; 3 Jahre Behandlung) zeigte einen signifikanten Unterschied bezüglich des kombinierten primären Endpunkts (Verdopplung des Serumkreatinins, Zeit bis zur Nierenersatztherapie oder Gesamtmortalität) zugunsten der Natriumhydrogenkarbonat- im Vergleich zur Standardbehandlungsgruppe [9]. Fehlende Verblindung und Standardisierung der Behandlung zwischen den Zentren sind als wesentliche Limitationen dieser Studie zu nennen. Die Resultate der untersuchten Studien sind nicht einheitlich. So untersuchte eine weitere Studie (BiCARB; multizentrisch; 300 geriatrische Patienten mit CKD und leichter MA; 2 Jahre Behandlung) die Auswirkungen von Bikarbonat auf die körperliche Leistungsfähigkeit [4]. Im Vergleich zu Placebo zeigte die Studie nach einem Jahr keinen Nutzen in Bezug auf die Nierenfunktion, einen sekundären Endpunkt. Eine wesentliche Limitation war jedoch das pragmatische Studiendesign, das häufige Wechsel zwischen den Behandlungsgruppen ermöglichte und in der Folge zu einer Angleichung der Serumbikarbonatwerte in den beiden Gruppen führte. Eine Pilotstudie (BASE Pilot Trial) untersuchte die Sicherheit, Verträglichkeit, Adhärenz und Pharmakodynamik von Natriumhydrogenkarbonat. Ziel dieser Studie war es, die Grundlage für eine groß angelegte Studie zu schaffen, welche auch moderate Behandlungseffekte nachweisen kann [28].

In der neuen KDIGO-Leitlinie wurde die Empfehlung zur Therapie einer metabolischen Azidose angepasst

Aufgrund der uneinheitlichen Studienergebnisse und methodischen Limitationen wurde in der aktuellen Kidney-Disease: Improving-Global-Outcomes(KDIGO)-Leitlinie von 2024 die Empfehlung zur Behandlung der MA bei CKD angepasst. Die Therapie wird nun erst ab einem Serumbikarbonatwert < 18 mmol/l empfohlen, im Vergleich zu dem zuvor geltenden Bereich von < 22 mmol/l [18]. Bei Patienten (≧ 1 Jahr) nach Nierentransplantation konnte in der bislang einzigen Interventionsstudie kein Vorteil einer 2‑jährigen Azidosekorrektur mit Natriumhydrogenkarbonat hinsichtlich der Transplantatfunktion gezeigt werden [24].

## Knochenstoffwechsel

Knochenerkrankungen bei CKD („CKD-mineral bone disorders“ [MBD]) umfassen ein breites Spektrum von Entitäten, unter anderem renale Osteodystrophie, Osteomalazie, adynamische Knochenerkrankung, Osteopenie und Osteoporose. Knochen dienen als wichtiger Puffer bei Säurebelastung, und das Risiko für Frakturen ist bei CKD erhöht [19]. Epidemiologische Daten aus den USA zeigten einen Zusammenhang zwischen niedrigem Serumbikarbonat und verringerter Knochendichte [8]. In einer kürzlichen Querschnittsanalyse von 180 Patienten mit CKD und MA waren Marker für Knochenumsatz erhöht [22]. Daten aus Interventionsstudien beim Menschen sind jedoch widersprüchlich. Eine kleine Studie bei postmenopausalen Frauen ohne CKD zeigte eine Besserung des Knochenstoffwechsels unter Kaliumbikarbonat, und eine kürzlich durchgeführte Studie bei Patienten mit CKD konnte eine Veränderung von Knochenstoffwechselparametern zeigen. Eine weitere Studie wies dagegen bei CKD keine positive Wirkung auf die Knochendichte des Schenkelhalses nach [23, 29, 32]. Nierentransplantatempfänger leiden häufig an einer Osteopenie oder Osteoporose, die nach der Transplantation fortschreitet [26]. In einer großen französischen Kohorte wurde kürzlich gezeigt, dass eine Azidämie (niedriger arterieller Blut-pH) bei Patienten mit Nierentransplantat und Serumbikarbonat < 22 mmol/l mit Veränderungen des Mineralstoffwechsels assoziiert ist [5]. Bislang gibt es jedoch nur eine kleine RCT unter Einschluss von 30 Patienten mit Nierentransplantat und MA. In dieser Studie wurden verschiedene Aspekte der Knochenqualität untersucht [33]. Die Knochenstruktur war in der Kaliumcitratgruppe im Vergleich zur Kontrollgruppe (Kaliumchlorid) nach 12 Monaten Behandlung besser erhalten. Die Tetracyclinmarkierung und serologische Marker deuteten auf einen stärkeren Knochenumsatz in der Alkaligruppe im Vergleich zur Kontrollgruppe hin. Interessanterweise war die Knochendichte jedoch nicht signifikant unterschiedlich.

## Kardiovaskuläre Effekte

Mehrere Beobachtungsstudien – wenn auch nicht alle – haben einen Zusammenhang zwischen dem Schweregrad der MA und kardiovaskulären Ereignissen festgestellt. Dazu gehören erhöhte Raten an Herzinsuffizienz, Hirninfarkten, Myokardinfarkten und kardiovaskulärem Tod [12, 31]. Der genaue Mechanismus dieser Risikoerhöhung ist bislang unklar. Es wird angenommen, dass eine MA die Expression proinflammatorischer Gene in Endothelzellen verstärkt und die Zelladhärenz zwischen Endothelzellen beeinträchtigt. Dies begünstigt die Infiltration von Leukozyten und den Austritt von Plasma, was zu Gewebeschäden führt. Darüber hinaus aktiviert eine MA das Renin-Angiotensin-Aldosteron-System (RAAS), was durch eine verminderte myokardiale Kontraktilität zur Herzinsuffizienz beitragen kann. Große Interventionsstudien, die den Effekt einer Alkalitherapie auf harte kardiovaskuläre Endpunkte untersuchen, fehlen bisher.

Zudem besteht die Befürchtung, dass der hohe Natriumgehalt vieler Alkalitherapien zu einer Volumenretention führen, den Blutdruck erhöhen und die kardioprotektiven Effekte einer RAAS-Blockade abschwächen könnte [14]. Drei Metaanalysen ergaben widersprüchliche Resultate bezüglich der Auswirkung von Natriumhydrogenkarbonat auf den Blutdruck; in zwei Metaanalysen wurden erhöhte systolische Blutdruckwerte gesehen [3, 27, 37]. Eine basenreiche Ernährung mit Früchten und Gemüse war im Vergleich zu Natriumhydrogenkarbonat in einer RCT über 5 Jahre bezüglich des Blutdrucks überlegen [13]. In einer kleinen unverblindeten Cross-over-Interventionsstudie (*n* = 20) konnte bei CKD und MA durch eine Bikarbonatsubstitution die Endothelfunktion signifikant verbessert werden [17]. Bei Patienten nach Nierentransplantation hat sich bislang kein negativer Effekt auf den Blutdruck gezeigt [24].

## Mortalität

In den meisten Studien war MA bei CKD mit einer erhöhten Mortalität assoziiert, sowohl vor als auch nach Beginn einer Hämodialyse und nach Transplantation [20, 30, 31, 34]. Eine CKD-Studie mit US-Veteranen zeigte, dass das Vorhandensein einer MA (Bikarbonat < 22 mmol/l) mit einer 43 % höheren Gesamtmortalität verbunden war. Die genauen Ursachen für dieses erhöhte Mortalitätsrisiko sind unklar, es wird jedoch eine indirekte Pathogenese über die oben genannten kardiovaskulären Ereignisse angenommen. Ob eine Normalisierung des Serumbikarbonats durch eine Alkalitherapie dieses Sterblichkeitsrisiko senken kann, ist noch nicht abschließend geklärt. In der bisher größten randomisierten CKD-Interventionsstudie konnte die Alkalitherapie die Gesamtmortalität um 57 % reduzieren, allerdings wies die Studie wesentliche Limitationen auf, die bereits weiter oben diskutiert wurden [9]. Dieser Mortalitätsvorteil konnte in einer kürzlich veröffentlichten Metaanalyse nicht bestätigt werden; eine Bikarbonatsupplementation zeigte in sechs RCT keinen Einfluss auf die Gesamtmortalität [37].

## Muskelabbau, Hypalbuminämie und Ernährungszustand

Der Zusammenhang zwischen MA, Muskelverlust und verminderter Albuminsynthese ist gut belegt. Das Ungleichgewicht zwischen Proteinsynthese und Proteinabbau führt zu einer negativen Proteinbilanz, die mit einem Verlust an Muskelmasse und -kraft einhergeht. Dies wiederum ist mit einer erhöhten Hospitalisierungsrate, schlechteren Lebensqualität und höheren Mortalität verbunden. Eine proteinreduzierte Diät, wie sie zur Verzögerung des Fortschreitens der CKD noch teilweise empfohlen wird, kann den Muskelkatabolismus zusätzlich verstärken. Eine Alkaliintervention zeigte signifikante Verbesserungen des Ernährungsstatus [6]. Die Zunahme an „lean body mass“ (gemessen anhand des Oberarmumfangs) wurde auch in einer Metaanalyse nachgewiesen [37]. Allerdings konnte für eine Alkalisubstitution in einer geriatrischen Population (≥ 60 Jahre) keine Verbesserung bezüglich Gleichgewicht, Gehgeschwindigkeit oder Beinkraft nachgewiesen werden [4].

## Endokrinologische Effekte

### Glukosestoffwechsel und Insulinresistenz

In der Literatur sind verschiedene Effekte einer MA auf die Insulinresistenz beschrieben [10]. Hierbei wurde gezeigt, dass die MA zu einem gestörten Glukosestoffwechsel sowie zu einer verminderten zellulären Insulinsensitivität führt. Des Weiteren wird durch eine MA die Kortisolproduktion gesteigert, die wiederum die Insulinsensitivität in den Muskel- und Fettzellen reduziert. Ebenfalls werden die Bindung von Insulin an den Insulinrezeptor und die darauffolgende Signalkaskade negativ beeinflusst. Obwohl es mehrere Studien bezüglich der Assoziation einer MA mit Insulinresistenz gibt, existiert leider bisher nur eine Interventionsstudie, die den Effekt einer Alkalitherapie auf das „homeostatic model assessment“ (HOMA) untersucht hat [1]. In dieser randomisierten Open-label-Studie (1:1 Standardtherapie vs. Natriumhydrogenkarbonat) konnte die Alkalitherapie zu einer geringeren Insulinresistenz gemäß HOMA führen, wobei die größte Reduktion bei einem Bikarbonatwert zwischen 24 und 28 mmol/l beschrieben wurde.

### Schilddrüsenfunktion

Bereits vor mehreren Dekaden konnte gezeigt werden, dass es im Rahmen einer MA zu einer verminderten Sekretion von Schilddrüsenhormonen sowie zu Veränderungen des Schilddrüsenstoffwechsels kommt [7]. Eine randomisierte Studie bei prädialytischen Patienten mit CKD (geschätzte glomeruläre Filtrationsrate < 35 ml/min) und MA (totales CO_2_ ≤ 22 mmol/l) konnte für die mit oralem Natriumhydrogenkarbonat behandelte Gruppe (Zielbereich Bikarbonat ≥ 24 mmol/l) eine signifikante Besserung der Schilddrüsenfunktion zeigen [11]. In derselben Studie war das Serumkalium in den beiden Gruppen ohne Unterschied. Eine weitere kleine Studie untersuchte bei 14 Patienten unter Hämodialyse den Effekt von Natriumcitrat vs. Natriumchlorid auf Schilddrüsenfunktion, Wachstumshormon, Glukokortikoidaktivität sowie Serumalbumin [36]. Hier kam es unter der 4‑wöchigen Alkalitherapie mit Natriumcitrat zu einem Anstieg von freiem Trijodthyronin (fT_3_) sowie zu einer Verbesserung von Glukokortikoidsensitivität und Serumalbumin. Im Frequent Hemodialysis Network Trial (FNH) wurden die Teilnehmer entweder mit konventioneller Hämodialyse (3-mal/Woche) im Vergleich zu einer häufigen Hämodialyse (6-mal/Woche, „daily trial“) oder mit einer nächtlichen Hämodialyse (3-mal/Woche) im Vergleich zu einer häufigen nächtlichen Hämodialyse („nocturnal trial“) behandelt [25]. Interessanterweise war ein Anstieg des Serumbikarbonats nicht mit einem Anstieg der Schilddrüsenhormone fT_3_ und freies Thyroxin (fT_4_) assoziiert.

## Fazit für die Praxis

- Die metabolische Azidose (MA) ist eine häufige Komplikation bei chronischer Nierenkrankheit („chronic kidney disease“ [CKD]) und nach Nierentransplantation.
- Eine MA scheint unter anderem negative Auswirkungen auf CKD-Progression, Knochenstoffwechsel, kardiovaskuläres System, Mortalität, Proteinkatabolismus und Insulinresistenz sowie Schilddrüsenfunktion zu haben – allerdings ist die Anzahl der randomisierten, kontrollierten Studien und somit auch die Evidenzlage hinsichtlich des Nutzens einer Alkalitherapie zurzeit noch sehr limitiert.
- Eine MA sollte insbesondere ab einem CKD-Stadium G3b (KDIGO) mithilfe einer venösen Blutgasanalyse abgeklärt werden. Eine Alkalitherapie sollte gemäß der aktuellen KDIGO-Leitlinie bei einem Serumbikarbonat < 18 mmol/l eingeleitet werden.
- Eine Korrektur der MA kann mittels pharmakologischer und diätetischer Maßnahmen angestrebt werden. In der Behandlung von Kindern können bei Bedarf flüssige Darreichungsformen erwogen werden.

## References

[1] Bellasi A, Di Micco L, Santoro D et al (2016) Correction of metabolic acidosis improves insulin resistance in chronic kidney disease. BMC Nephrol 17:158. 10.1186/s12882-016-0372-x 27770799 10.1186/s12882-016-0372-x PMC5075179

[2] Bertholet-Thomas A, Guittet C, Manso-Silván MA et al (2021) Efficacy and safety of an innovative prolonged-release combination drug in patients with distal renal tubular acidosis: an open-label comparative trial versus standard of care treatments. Pediatr Nephrol 36:83–91. 10.1007/s00467-020-04693-2 32712761 10.1007/s00467-020-04693-2 PMC7701073

[3] Beynon-Cobb B, Louca P, Hoorn EJ et al (2023) Effect of Sodium Bicarbonate on Systolic Blood Pressure in CKD: A Systematic Review and Meta-Analysis. Clin J Am Soc Nephrol 18:435–445. 10.2215/CJN.0000000000000119 36758154 10.2215/CJN.0000000000000119 PMC10103210

[4] BiCARB study group (2020) Clinical and cost-effectiveness of oral sodium bicarbonate therapy for older patients with chronic kidney disease and low-grade acidosis (BiCARB): a pragmatic randomised, double-blind, placebo-controlled trial. BMC Med 18:91. 10.1186/s12916-020-01542-9 32268897 10.1186/s12916-020-01542-9 PMC7144058

[5] Brazier F, Jouffroy J, Martinez F et al (2020) Association of blood bicarbonate and pH with mineral metabolism disturbance and outcome after kidney transplantation. Am J Transplant 20:1063–1075. 10.1111/ajt.15686 31680427 10.1111/ajt.15686

[6] de Brito-Ashurst I, Varagunam M, Raftery MJ et al (2009) Bicarbonate supplementation slows progression of CKD and improves nutritional status. J Am Soc Nephrol 20:2075–2084. 10.1681/ASN.2008111205 19608703 10.1681/ASN.2008111205 PMC2736774

[7] Brüngger M, Hulter HN, Krapf R (1997) Effect of chronic metabolic acidosis on thyroid hormone homeostasis in humans. Am J Physiol 272:F648–653. 10.1152/ajprenal.1997.272.5.F648 9176376 10.1152/ajprenal.1997.272.5.F648

[8] Chen W, Melamed ML, Abramowitz MK (2015) Serum bicarbonate and bone mineral density in US adults. Am J Kidney Dis 65:240–248. 10.1053/j.ajkd.2014.07.007 25168294 10.1053/j.ajkd.2014.07.007 PMC4305466

[9] Di Iorio BR, Bellasi A, Raphael KL et al (2019) Treatment of metabolic acidosis with sodium bicarbonate delays progression of chronic kidney disease: the UBI study. J Nephrol 32:989–1001. 10.1007/s40620-019-00656-5 31598912 10.1007/s40620-019-00656-5 PMC6821658

[10] DiNicolantonio JJ, O’Keefe JH (2021) Low-grade metabolic acidosis as a driver of insulin resistance. Open Heart 8:e1788. 10.1136/openhrt-2021-001788 34497064 10.1136/openhrt-2021-001788 PMC8438953

[11] Disthabanchong S, Treeruttanawanich A (2010) Oral sodium bicarbonate improves thyroid function in predialysis chronic kidney disease. Am J Nephrol 32:549–556. 10.1159/000321461 21042013 10.1159/000321461

[12] Dobre M, Pajewski NM, Beddhu S et al (2020) Serum bicarbonate and cardiovascular events in hypertensive adults: results from the Systolic Blood Pressure Intervention Trial. Nephrol Dial Transplant 35:1377–1384. 10.1093/ndt/gfz149 32163578 10.1093/ndt/gfz149 PMC7462723

[13] Goraya N, Madias NE, Simoni J et al (2024) Kidney and Cardiovascular Protection Using Dietary Acid Reduction in Primary Hypertension: A Five-Year, Interventional, Randomized, Control Trial. Am J Med. 10.1016/j.amjmed.2024.06.006 (S0002–9343(24)00357–7) 39107215 10.1016/j.amjmed.2024.06.006

[14] Heerspink HJL, Holtkamp FA, Parving H‑H et al (2012) Moderation of dietary sodium potentiates the renal and cardiovascular protective effects of angiotensin receptor blockers. Kidney Int 82:330–337. 10.1038/ki.2012.74 22437412 10.1038/ki.2012.74

[15] Hultin S, Hood C, Campbell KL et al (2021) A Systematic Review and Meta-Analysis on Effects of Bicarbonate Therapy on Kidney Outcomes. Kidney Int Rep 6:695–705. 10.1016/j.ekir.2020.12.019 33732984 10.1016/j.ekir.2020.12.019 PMC7938083

[16] Inker LA, Grams ME, Levey AS et al (2019) Relationship of Estimated GFR and Albuminuria to Concurrent Laboratory Abnormalities: An Individual Participant Data Meta-analysis in a Global Consortium. Am J Kidney Dis 73:206–217. 10.1053/j.ajkd.2018.08.013 30348535 10.1053/j.ajkd.2018.08.013 PMC6348050

[17] Kendrick J, Shah P, Andrews E et al (2018) Effect of Treatment of Metabolic Acidosis on Vascular Endothelial Function in Patients with CKD: A Pilot Randomized Cross-Over Study. Clin J Am Soc Nephrol 13:1463–1470. 10.2215/CJN.00380118 30237219 10.2215/CJN.00380118 PMC6218835

[18] Kidney Disease: Improving Global Outcomes (KDIGO) CKD Work Group (2024) KDIGO 2024 Clinical Practice Guideline for the Evaluation and Management of Chronic Kidney Disease. Kidney Int 105:S117–S314. 10.1016/j.kint.2023.10.018 38490803 10.1016/j.kint.2023.10.018

[19] Kim SM, Long J, Montez-Rath M et al (2016) Hip Fracture in Patients With Non-Dialysis-Requiring Chronic Kidney Disease. J Bone Miner Res 31:1803–1809. 10.1002/jbmr.2862 27145189 10.1002/jbmr.2862

[20] Kovesdy CP, Anderson JE et al (2008) Association of serum bicarbonate levels with mortality in patients with non-dialysis-dependent CKD. Nephrol Dial Transplant 24:1232–1237. 10.1093/ndt/gfn633 19015169 10.1093/ndt/gfn633 PMC2721428

[21] Kuhn C, Mohebbi N, Ritter A (2024) Metabolic acidosis in chronic kidney disease: mere consequence or also culprit? Pflugers Arch 476:579–592. 10.1007/s00424-024-02912-5 38279993 10.1007/s00424-024-02912-5 PMC11006741

[22] Levy RV, McMahon DJ, Agarwal S et al (2023) Comprehensive Associations between Acidosis and the Skeleton in Patients with Kidney Disease. J Am Soc Nephrol 34:668–681. 10.1681/ASN.0000000000000085 36749125 10.1681/ASN.0000000000000085 PMC10103353

[23] Melamed ML, Horwitz EJ, Dobre MA et al (2020) Effects of Sodium Bicarbonate in CKD Stages 3 and 4: A Randomized, Placebo-Controlled, Multicenter Clinical Trial. Am J Kidney Dis 75:225–234. 10.1053/j.ajkd.2019.07.016 31699517 10.1053/j.ajkd.2019.07.016 PMC7012673

[24] Mohebbi N, Ritter A, Wiegand A et al (2023) Sodium bicarbonate for kidney transplant recipients with metabolic acidosis in Switzerland: a multicentre, randomised, single-blind, placebo-controlled, phase 3 trial. Lancet 401:557–567. 10.1016/S0140-6736(22)02606-X 36708734 10.1016/S0140-6736(22)02606-X

[25] Molfino A, Beck GJ, Li M et al (2019) Association between change in serum bicarbonate and change in thyroid hormone levels in patients receiving conventional or more frequent maintenance haemodialysis. Nephrology 24:81–87. 10.1111/nep.13187 29064128 10.1111/nep.13187 PMC5916580

[26] Molnar MZ, Naser MS, Rhee CM et al (2014) Bone and mineral disorders after kidney transplantation: therapeutic strategies. Transplant Rev 28:56–62. 10.1016/j.trre.2013.12.003 10.1016/j.trre.2013.12.003 PMC5588857 24462303

[27] Navaneethan SD, Shao J, Buysse J et al (2019) Effects of Treatment of Metabolic Acidosis in CKD: A Systematic Review and Meta-Analysis. Clin J Am Soc Nephrol 14:1011–1020. 10.2215/CJN.13091118 31196951 10.2215/CJN.13091118 PMC6625635

[28] Raphael KL, Isakova T, Ix JH et al (2020) A randomized trial comparing the safety, adherence, and pharmacodynamics profiles of two doses of sodium bicarbonate in CKD: the BASE pilot trial. J Am Soc Nephrol 31:161–174. 10.1681/ASN.2019030287 31848294 10.1681/ASN.2019030287 PMC6934992

[29] Raphael KL, Katz R, Larive B et al (2024) Oral Sodium Bicarbonate and Bone Turnover in CKD: A Secondary Analysis of the BASE Pilot Trial. J Am Soc Nephrol 35:57–65. 10.1681/ASN.0000000000000264 38170601 10.1681/ASN.0000000000000264 PMC10786609

[30] Raphael KL, Zhang Y, Wei G et al (2013) Serum bicarbonate and mortality in adults in NHANES III. Nephrol Dial Transplant 28:1207–1213. 10.1093/ndt/gfs609 23348878 10.1093/ndt/gfs609 PMC3808693

[31] Ritter A, Mohebbi N (2020) Causes and Consequences of Metabolic Acidosis in Patients after Kidney Transplantation. Kidney Blood Press Res 45:792–801. 10.1159/000510158 33040055 10.1159/000510158

[32] Sebastian A, Harris ST, Ottaway JH et al (1994) Improved mineral balance and skeletal metabolism in postmenopausal women treated with potassium bicarbonate. N Engl J Med 330:1776–1781. 10.1056/NEJM199406233302502 8190153 10.1056/NEJM199406233302502

[33] Starke A, Corsenca A, Kohler T et al (2012) Correction of metabolic acidosis with potassium citrate in renal transplant patients and its effect on bone quality. Clin J Am Soc Nephrol 7:1461–1472. 10.2215/CJN.01100112 22773591 10.2215/CJN.01100112 PMC3430948

[34] Tangri N, Reaven NL, Funk SE et al (2021) Metabolic acidosis is associated with increased risk of adverse kidney outcomes and mortality in patients with non-dialysis dependent chronic kidney disease: an observational cohort study. BMC Nephrol 22:185. 10.1186/s12882-021-02385-z 34011303 10.1186/s12882-021-02385-z PMC8136202

[35] Trepiccione F, Walsh SB, Ariceta G et al (2021) Distal renal tubular acidosis: ERKNet/ESPN clinical practice points. Nephrol Dial Transplant 36:1585–1596. 10.1093/ndt/gfab171 33914889 10.1093/ndt/gfab171

[36] Wiederkehr MR, Kalogiros J, Krapf R (2004) Correction of metabolic acidosis improves thyroid and growth hormone axes in haemodialysis patients. Nephrol Dial Transplant 19:1190–1197. 10.1093/ndt/gfh096 14993483 10.1093/ndt/gfh096

[37] Yang T‑Y, Lin H‑M, Wang H‑Y et al (2024) Sodium Bicarbonate Treatment and Clinical Outcomes in Chronic Kidney Disease with Metabolic Acidosis: A Meta-Analysis. Clin J Am Soc Nephrol 19:959–969. 10.2215/CJN.0000000000000487 38980732 10.2215/CJN.0000000000000487 PMC11321727

